# Genetic and clinical features of Chinese sporadic amyotrophic lateral sclerosis patients with *TARDBP* mutations

**DOI:** 10.1002/brb3.2312

**Published:** 2021-08-01

**Authors:** Feng Feng, Hongfen Wang, Jiajin Liu, Zhanjun Wang, Baixuan Xu, Kun Zhao, Xiaoyong Tao, Zhengqing He, Fei Yang, Xusheng Huang

**Affiliations:** ^1^ Department of Neurology, First Medical Center Chinese PLA General Hospital Beijing China; ^2^ Department of Neurology PLA Rocket Force Characteristic Medical Center Beijing China; ^3^ Department of Nuclear Medicine, First Medical Center Chinese PLA General Hospital Beijing China; ^4^ Department of Neurology, Xuanwu Hospital Capital Medical University Beijing China; ^5^ School of Biological Science and Medical Engineering Beihang University Beijing China; ^6^ Department of Neurology, Eighth Medical Center Chinese PLA General Hospital Beijing China

**Keywords:** Chinese, frontotemporal dementia, semantic variant primary progressive aphasia, sporadic amyotrophic lateral sclerosis, TARDBP

## Abstract

**Objectives:**

To investigate the genetic and clinical features of Chinese sporadic amyotrophic lateral sclerosis (SALS) patients with *TARDBP* mutations, we carried out a genetic analysis in a cohort of 391 SALS patients and explored the clinical manifestations of patients with *TARDBP* variants.

**Materials and methods:**

The coding region of all five coding exons of *TARDBP*, exons 2–6, were sequenced for mutations in 391 Chinese SALS patients. The clinical features of patients with *TARDBP* mutations were described and compared with cases in literatures.

**Results:**

Two missense mutations in *TARDBP* gene, c.1132A > G (p.N378D) and c.1147A > G (p.I383V), were detected in three cases, showing a low frequency (0.77%, 3/391) of *TARDBP* missense mutations in Chinese SALS patients. Based on a retrospective analysis of literatures, p.N378D mutation mainly presents a phenotype of early onset, whereas p.I383V mutation presents pure ALS or ALS alongside semantic variant primary progressive aphasia (svPPA), a type of frontotemporal dementia (FTD).

**Conclusions:**

Our results demonstrate that *TARDBP* mutation is a rare cause of Chinese SALS patients and expand the spectrum of phenotype. It is implied that genetic analysis of SALS patients plays a crucial role in uncovering the cause of disease, especially for cases developing early onset or alongside FTD.

## Graphical abstract

As an overlapping gene related to ALS and FTD, TARDBP gene detection is especially necessary for ALS patients with FTD or early onset ALS cases. The sequence of TARDBP gene is bound to provide more perspectives for understanding the pathogenesis of SALS.

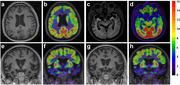

## 1 INTRODUCTION

Amyotrophic lateral sclerosis (ALS) is a progressive neurodegenerative disease and mainly presents muscle atrophy, weakness, fasciculation and hyperreflexia. ALS patients mostly die of respiratory failure within 3–5 years after disease onset. Approximately 5–10% of ALS cases are familial ALS (FALS), and the remaining cases are sporadic ALS (SALS) (Kiernan et al., 2011). In addition, 5% of ALS patients may comorbid frontotemporal dementia (FTD) (Kiernan et al., 2011). Although the etiology of ALS remains unclear, gene mutations, especially in *SOD1*, *C9orf72*, *FUS*, and *TARDBP* (Mathis et al., 2019), account for a majority of FALS and a minority of SALS cases.

*TARDBP* gene encodes transactive response DNA‐binding protein 43 (TDP‐43), a critical protein for RNA regulation, transcription, and translation. Under pathological conditions, TDP‐43 translocates to cytoplasm from nuclei and develops the ubiquitin‐positive but tau‐negative inclusions, a pathological hallmark of ALS and FTD with ubiquitin‐positive inclusions. To date, mutations in *TARDBP* gene associated with ALS has exceeded 60 on the Human Gene Mutation Database (HGMD). Correspondingly, clinical features of ALS patients with *TARDBP* mutations are heterogeneous. A review of 205 ALS patients with *TARDBP* variants showed that the mean age of onset was 57.3 years old, while spinal, bulbar and other onset accounted for 44.0%, 23.9%, and 32.2% of cases, respectively (Connolly et al., 2020). Only 12% of ALS cases with *TARDBP* variants were reported alongside FTD (Connolly et al., 2020).

A meta‐analysis showed that 3.3% of FALS patients and 0.5% of SALS patients harbored *TARDBP* mutations (Zou et al., 2017). In Chinese ALS patients, *TARDBP* mutations accounted for 5.8% FALS cases and 0.3% SALS cases (Wei et al., 2019). Although the frequency of *TARDBP* mutations is much lower in SALS than in FALS, it is still an important cause of SALS that cannot be ignored. In the present study, we screened *TARDBP* gene in a large cohort of Chinese SALS patients to investigate the clinical features of SALS patients with *TARDBP* mutations. Furthermore, we reviewed and summarized SALS cases with the detected mutations in literatures to explore the correlations between genotype and phenotype.

## 2 MATERIALS AND METHODS

### 2.1 Participants enrollment

A total of 391 Chinese patients meeting the revised El Escorial criteria (Brooks et al., 2000) for clinically definite, clinically probable or clinically possible SALS were included in the study. They were all from Chinese Han populations, as well as *SOD1* and *FUS* gene negative. SALS is defined as without family history of ALS or FTD. If the patient had at least one first‐degree or second‐degree relative with ALS or FTD, the family history was considered positive. These participants were inpatients and outpatients in the Department of Neurology of the First Medical Center, Chinese PLA General Hospital, from 2015 to 2021.

Clinical characteristics, including the age of onset, site of onset, duration, results of neurological examination and electromyography (EMG), were analyzed in detail. For SALS patients with cognitive impairment or abnormal behavior, Mini‐Mental State Examination (MMSE), Montreal Cognitive Assessment (MoCA) and Edinburgh Cognitive and Behavioural ALS Screen (ECAS) were completed by a senior neurologist, in spite of neuroimaging examination. Diagnosis of FTD was made in line with the revised criteria (Strong et al., 2017). This study was approved by the Medical Ethics Committee of the Chinese PLA General Hospital. All of the participants signed a consent form by themselves or their legal guardians.

### 2.2 Genetic analysis

Genomic DNA was extracted from peripheral blood leucocytes using a QIAamp Blood Midi Kit (QIAGEN, Germany) in strict accordance with the instructions. The coding region of all five coding exons of *TARDBP* (NM_007375), exons 2–6, and their exon–intron junctions (50 bp) were amplified by polymerase chain reaction (PCR) on an ABI 2720 (Applied Biosystems). The PCR products were subjected to direct Sanger sequencing on an ABI 3730 DNA Analyzer (Applied Biosystems) and analyzed for matches using Sequencing Analysis 5.2.0.

The pathogenicity of detected missense mutations was evaluated according to the American College of Medical Genetics and Genomics (ACMG) recommendations. The minor allele frequency (MAF) of detected missense mutations was investigated in the Exome Aggregation Consortium (ExAC) database (https://exac.broadinstitute.org/) and the Genome Aggregation Database (gnomAD; https://gnomad.broadinstitute.org).

Furthermore, patients with pathogenic or likely pathogenic *TARDBP* missense mutations underwent next‐generation sequencing (NGS) with an 80‐gene panel including reported ALS causative genes, FTD‐related genes and important genes relevant to differential diagnosis. Although the hexanucleotide (GGGGCC) repeat expansion within *C9orf72* gene is rare in Asian populations, it was also investigated in this study in patients with *TARDBP* mutations. Additionally, cosegregation of the detected *TARDBP* mutations was investigated within the family members who consented to genetic analysis.

## 3 RESULTS

### 3.1 Demographic and clinical characteristics

For 391 included SALS patients, the mean age of onset was 52.7 ± 10.7 years old and the ratio of males to females was 235:156. Among these patients, spinal‐onset and bulbar‐onset cases accounted for 80.3% (314/391) and 19.7% (77/391), respectively. The mean onset age of spinal‐onset group (51.9 ± 10.5 years old) was earlier than that of bulbar‐onset group (56.1 ± 11.0 years old) (*p *< .01), and the gender ratio (Male/Female) in the spinal‐onset group (200/114) was higher than that in the bulbar‐onset group (35/42) (*p *< .01) (Table S1).

According to the revised El Escorial criteria (Brooks et al., 2000), all of the included SALS patients were classified into clinically definite, clinically probable and clinically possible SALS. The demographic and clinical features for each diagnostic category of SALS patients were displayed in Table S2. In addition, there were seven SALS patients (Male/Female: 6/1) comorbid FTD before or after ALS onset (ALS‐FTD) in our cohort. Among them, five cases were spinal‐onset ALS (Male/Female: 5/0) and two cases were bulbar‐onset ALS (Male/Female: 1/1). The onset age of these seven ALS‐FTD patients ranged from 45 to 65 years, with a mean age of 55.4 ± 6.5 years.

### 3.2 Genetic analysis

Sequence analysis of 2‐6 exons of the *TARDBP* gene among 391 SALS patients identified two reported missense mutations (p.N378D and p.I383V, Figure S1) in three patients (Table 1), showing a 0.77% (3/391) frequency of *TARDBP* variants. In the above three patients, no pathogenic or likely pathogenic missense mutation was detected in the 80‐gene panel, and the number of hexanucleotide (GGGGCC) repeat expansion in *C9orf72* gene was normal. The p.N378D and p.I383V mutations in *TARDBP* were reported by Tsai et al. (2011) and by Rutherford et al. (2008), respectively. In this study, pathogenic analysis according to the ACMG recommendations demonstrated that p.N378D variant is pathogenic, whereas p.I383V mutation is likely pathogenic. In ExAC database and gnomAD, the MAF of p.N378D mutation is both unavailable, while the MAF of p.I383V mutation is 0.00008059 and 0.00006667, respectively. In addition, two synonymous substitutions in *TARDBP* gene, c.57A > G (p.P19P) and c.1098C > G (p.A366A), were also detected in this study.

**TABLE 1:** Genotypes and characteristics of Chinese SALS patients with TARDBP missense mutations in references and our cohort

	Nucleotide change	Amino acid change	Gender	AOO (years)	SOO	FTD	Duration (months)	Reference
1 patient	c.1133A > G	p.N378S	M	39	UL	No	>67	(Huang et al., 2012)
3 patients	c.875G > A	p.S292N	M	59	UL	No	38	(Zou et al., 2016)
	c.875G > A	p.S292N	M	52	B	No	35	
	c.1043G > T	p.G348V	F	36	UL	No	>54	
1 patient	c.1178C > T	p.S393L	M	58	UL	Yes	84	(Ju et al., 2016)
1 patient	c.1069G > A	p.G357S	M	65	UL	**—**	>24	(Liu et al., 2019)
3 patients	c.892G > A	p.G298S	**—**	**—**	**—**	No	**—**	(Chen et al., 2020)
	c.1123G > A	p.S375G						
	c.1112A > G	p.N371S						
4 patients	c.881G > T	p.G294V	M	61	B+UL	No	6	(Wang et al., 2020)
	c.881G > T	p.G294V	M	53	B	No	>18	
	c.1043G > T	p.G348V	M	24	UL	No	>120	
	c.1147A > G	p.I383V	F	45	UL	No	36	
Patient 1	c.1132A > G	p.N378D	F	39	UL	No	>24	This study
Patient 2	c.1147A > G	p.I383V	F	53	B	No	23	This study
Patient 3	c.1147A > G	p.I383V	M	57	LL	Yes	>61	This study

**Abbreviations: —** = absent; AOO = age of onset; B = bulbar; FTD = frontotemporal dementia; LL = lower limb; SOO = site of onset; UL = upper limb.

### 3.3 Clinical features of the patient with the TARDBP c.1132A > G (p. N378D) mutation

#### 3.3.1 Patient 1

**FIGURE 1:**
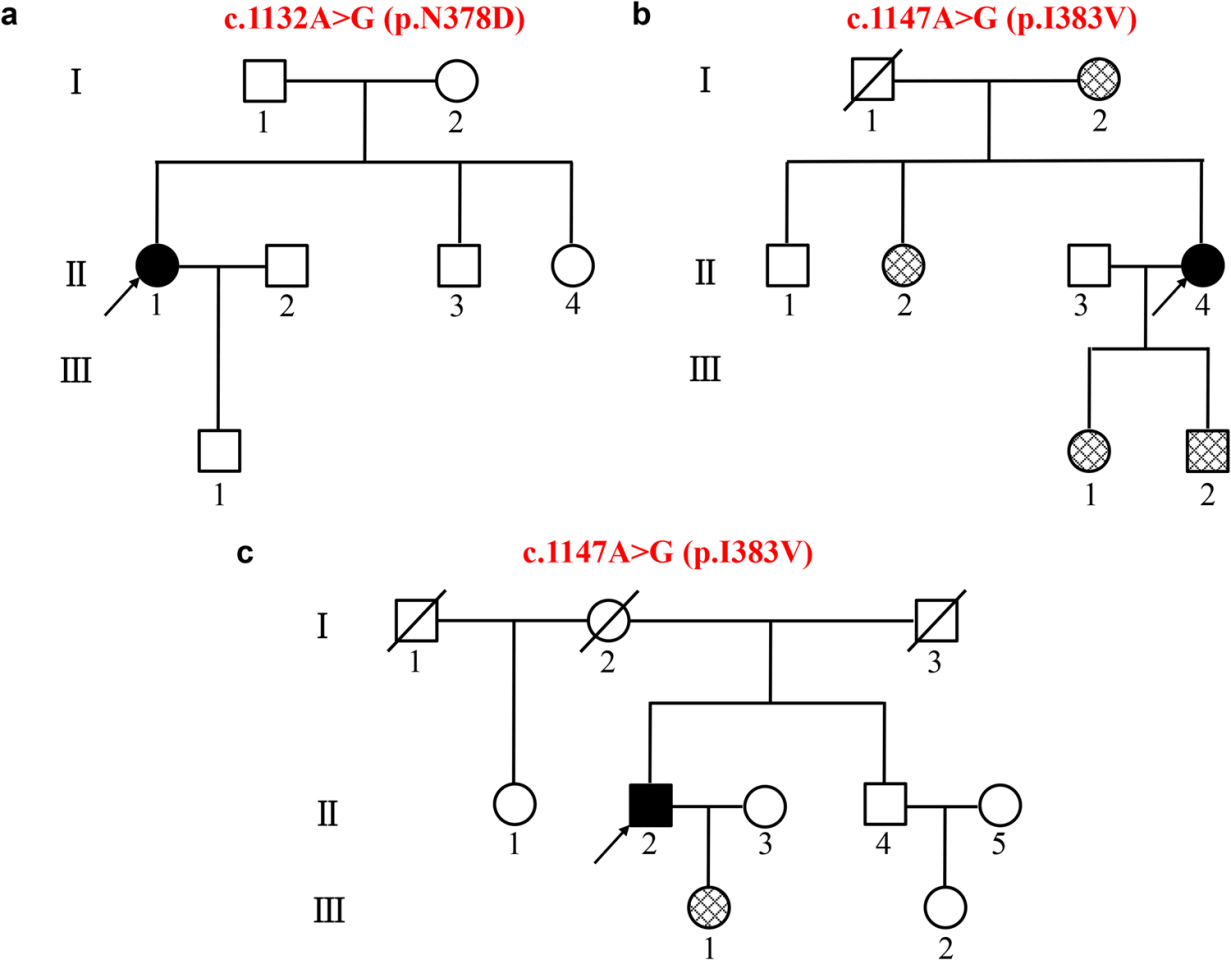
Family trees and genetic analysis of three patients with *TARDBP* mutations Family tree of patient 1 (a), patient 2 (b), and patient 3 (c) and results of genetic analysis. The black symbol represents the proband and the crosshatched symbol represents normal family members carrying the same mutation.

Beginning at 39 years of age, patient 1 (II‐1 in Figure 1a) developed weakness in the right hand and had difficulty feeding herself. Two months later, the weakness progressed to the proximal right limb, and the patient had difficulty washing and dressing herself. At the same time, atrophy of the right limb and fasciculation in the muscles both presented. One month later, she went to our clinic, and generalized hyperreflexia was found on neurological examination. EMG showed fibrillation potentials, positive sharp waves and long duration, high amplitude motor unit potentials in muscles of upper limbs. At that time, the patient was diagnosed with clinically possible ALS. Unfortunately, neuropsychological assessments were not carried out for the patient. Genetic test of her parents, younger brother and sister were not accomplished due to loss of follow‐up. However, similar symptoms or cognitive impairment in family members were denied by the patient.

### 3.4 Clinical features of the patients with the TARDBP c.1147A > G (p.I383V) mutation

#### 3.4.1 Patient 2

At the age of 53 years, patient 2 (II‐4 in Figure 1b) began to present dysarthria. Three months later, she exhibited obvious dysphagia and felt weakness in both hands. As the disease progressed, she also sensed fasciculation in her tongue, hands and legs. One month later, she felt mild dyspnea in a supine position and went to our clinic. The neurological examination demonstrated dysarthria, weakness, and atrophy of the lingualis and both hands, as well as generalized hyperreflexia. EMG showed fibrillation potentials, positive sharp waves, and long duration, high‐amplitude motor unit potentials in muscles innervated by brainstem and all the spinal cord segments. Ultimately, she was diagnosed with clinical definite ALS. Unfortunately, patient 2 did not complete the neuropsychological assessments due to her dysarthria and weakness of hands, but she had no cognitive impairment or abnormal behavior reported by herself or her caregivers. Twenty‐three months later, she died suddenly.

Test of first‐degree relatives revealed that her mother, sister, daughter, and son all had the same heterozygous mutation, while her brother did not. Her sister, brother, daughter, and son all had no any symptoms of ALS or FTD. Her mother presented cognitive impairment in her sixties and existed for more than 20 years. Although her mother did not accept a neurological examination in our clinic, manifestations of ALS was denied by her caregivers. Due to the slow progress of cognitive impairment, long duration, and absence of abnormal behavior, diagnosis of FTD for her mother was not supported.

#### 3.4.2 Patient 3

When he was 57 years old, patient 3 (II‐2 in Figure 1c) developed lack of concentration and difficulty in finding and understanding words, such as he could say “vegetable” but could not name a specific vegetable. His vocabulary continued to decline, which lead to the description difficulty with unaffected fluency. Three years later, he became irritable and even dropped objects. At the same time, he gradually developed signs of memory decline such as forgetting the names of friends, and he became less communicative with others. Two months later, he presented weakness, atrophy and fasciculation in the left foot, left thigh and right upper limb in sequence. After a month, he felt weakness in the right leg and preferred sweet foods, especially sugar. Half a month before he visited our clinic, he began hiding sweet foods, making impulsive purchases and rejecting attempts to correct his behavior, and his left upper limb weakened as well.

**FIGURE 2:**
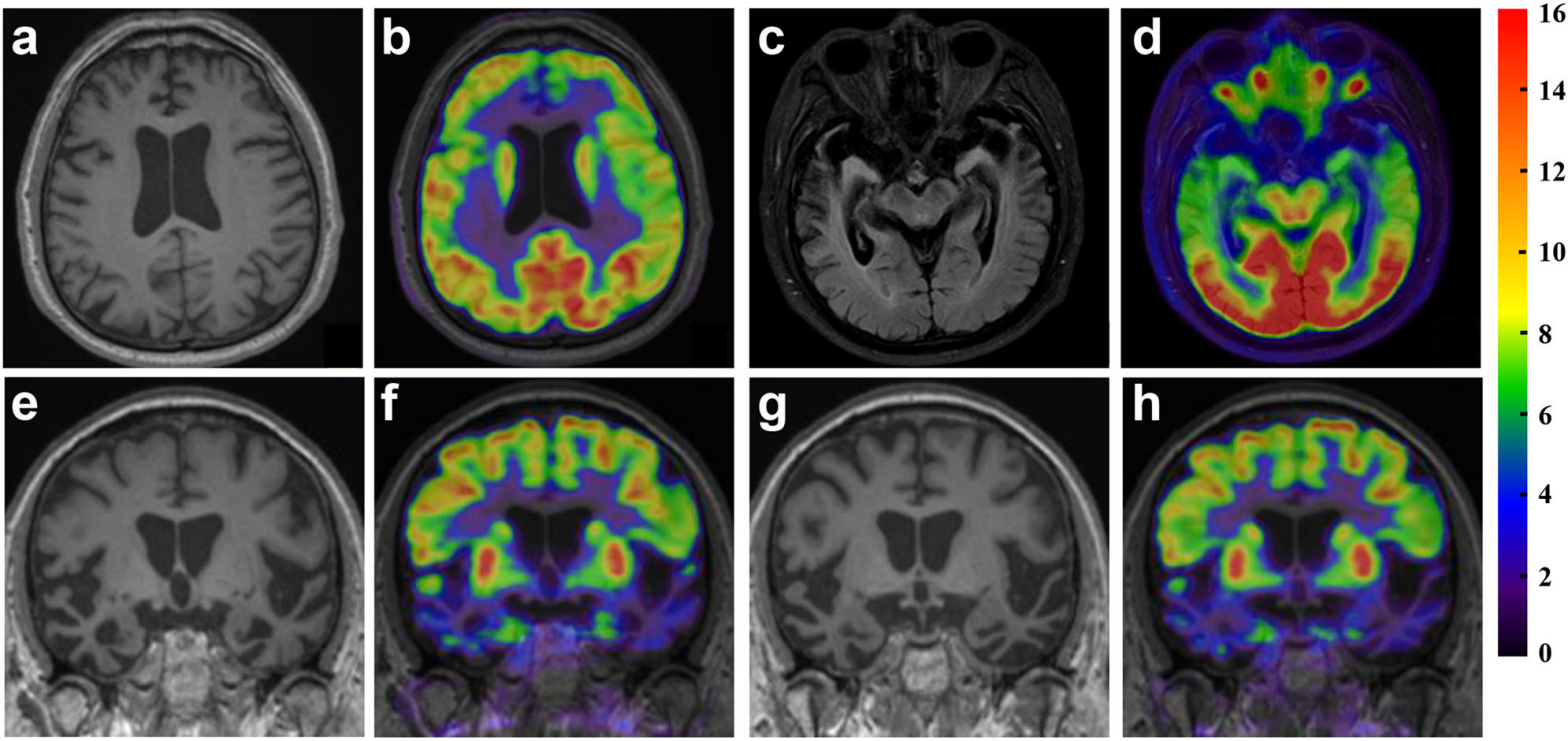
^18^F‐FDG PET/MR images of patient 3 Note the obvious hypometabolism, predominantly on the left side, without distinct atrophy in the bilateral frontal lobes (a, b), and severe, “blade‐like” atrophy in the bilateral temporal lobes along with significant hypometabolism, especially in the anterior and lateral parts (c–h)

Detailed neurological examination revealed mild‐to‐severe weakness and atrophy in all of the limbs, especially in the lower limbs, as well as generalized hyperreflexia. EMG demonstrated fibrillation potentials, positive sharp waves and long duration, high amplitude motor unit potentials in muscles innervated by spinal cord, and a diagnosis of clinically definite ALS was made accordingly. With 6 years of education, he scored 19/30 on the MMSE and 14/30 on the MoCA but did not complete the ECAS. Furthermore, ^18^F‐fluorodeoxyglucose (^18^F‐FDG) PET/MR examination was carried out in our hospital with a PET/MR hybrid system (Siemens, Biograph mMR). Severe atrophy was found in the bilateral temporal lobes, along with significant hypometabolism; obvious hypometabolism was seen in the bilateral frontal lobes (Figure 2). Thus, a diagnosis of semantic variant primary progressive aphasia (svPPA), a type of FTD, was made in accordance with the international consensus criteria (Gorno‐Tempini et al., 2011).

Sanger sequencing of the locus was performed for his daughter, his brother as well as daughter of his brother. Only his daughter carried the same mutation, but she had no any symptoms of ALS or FTD.

## 4 DISCUSSION

In our cohort, a total of 391 Chinese SALS patients from Han populations were screened for mutations in *TARDBP* gene. Two missense mutations, c.1132A > G (p.N378D) and c.1147A > G (p.I383V), were identified in three patients.

The frequency of *TARDBP* missense mutations in the current cohort was 0.77% (3/391), resembling previous results of 0.61% (1/165) (Huang et al., 2012), 0.93% (3/324) (Zou et al., 2016), 0.50% (1/200) (Ju et al., 2016), and 1.19% (3/253) (Chen et al., 2020), except for 4.76% (1/21) (Liu et al., 2019), and 2.74% (4/146) (Wang et al., 2020) in Chinese SALS patients. At the same time, several previous studies did not identify any Chinese SALS patients with *TARDBP* missense mutations (Deng et al., 2019; Hou et al., 2016; Lin et al., 2020; Liu et al., 2016; Liu et al., 2017; Soong et al., 2014; Xiong et al., 2010; Xu et al., 2018; Ye et al., 2013). To date, altogether 16 studies including the present study, have been carried out to screen for *TARDBP* variants in SALS patients of Chinese origin (Chen et al., 2020; Deng et al., 2019; Hou et al., 2016; Huang et al., 2012; Ju et al., 2016; Lin et al., 2020; Liu et al., 2019; Liu et al., 2016; Liu et al., 2017; Soong et al., 2014; Wang et al., 2020; Xiong et al., 2010; Xu et al., 2018; Ye et al., 2013; Zou et al., 2016), and only 16 patients with a total of 11 distinct *TARDBP* missense mutations were identified among 2631 Chinese SALS patients (Table 1), implying a frequency of 0.61% (16/2631). Thus, it is evident that *TARDBP* missense mutation is a rare reason for Chinese SALS patients. However, *TARDBP* mutation frequency is distinct in different ethnicities (Zou et al., 2017). The frequency of *TARDBP* mutations is 2.7% in SALS patients from Italy (Lattante et al., 2012), 2.1% in those from France (Daoud et al., 2009), 0.4% in those from Japan (Iida et al., 2012), and zero in those from Korea (Kwon et al., 2012), implying *TARDBP* mutations may be more frequent in European SALS patients.

For patients with *TARDBP* missense mutations in Table 1, the average age of onset was 49.3 ± 11.4 years old. Also, an earlier age of onset was identified in French ALS patients with *TARDBP* mutations than in those without (Corcia et al., 2012). Although 51.3% of Caucasian patients with *TARDBP* mutations were upper limb onset, 58.8% of Asian patients with *TARDBP* mutations were bulbar onset (Corcia et al., 2012), the most frequent site of onset in the current study was still upper limb (Table 1). In addition, ALS patients with *TARDBP* mutations presented a longer duration (Corcia et al., 2012). In Table 1, the longest duration was more than 10 years, whereas the shortest was only 6 months. Even in SALS patients with the same missense mutation, duration also varied widely (Table 1). Last, despite one patient with p.S393L (Ju et al., 2016) and one patient with p.I383V in our study presented ALS with FTD, the majority of Chinese SALS patients with *TARDBP* missense mutations displayed a phenotype of pure ALS (Table 1).

The p.N378D mutation in *TARDBP* was first described in a male FALS patient from a Chinese Taiwan family in 2011 (Tsai et al., 2011). His daughter was also diagnosed with ALS and died at 34 years old, 2 years after the onset of symptoms in hand muscles. In 2019, the second FALS patient with p.N378D variant was reported in a Mainland Chinese family (Deng et al., 2019). This patient developed dysphagia when she was 33 years old. Her sister died at 24 years of age due to similar symptoms. In our cohort, patient 1 also had a p.N378D mutation in *TARDBP* but without a family history of ALS. Unfortunately, due to loss of follow‐up, patient 1 did not undergo a pedigree analysis for co‐segregation. Although the male FALS patient reported by Tsai (Tsai et al., 2011) did not present weakness until the age of 60 years, his daughter, two patients from the family described by Deng (Deng et al., 2019) and patient 1 in the present study all developed symptoms of ALS before the age of 40 years. It is implied that p.N378D variant tends to present with an early‐onset phenotype. Additionally, spinal onset and bulbar onset were both found in ALS patients with p.N378D variant, and none of them had symptoms of FTD (Table 2).

**TABLE 2:** Clinical features of ALS patients with p.N378D mutation in TARDBP

	Gender	AOO (years)	SOO	FTD	Duration (months)	Family history	Ethnicity	Reference
1 patient	M	60	LL	No	27	Yes	Chinese	(Tsai et al., 2011)
1 patient	F	33	B	**—**	**—**	Yes	Chinese	(Deng et al., 2019)
Patient 1	F	39	UL	No	>24	No	Chinese	This study

**Abbreviations: —** = absent; AOO = age of onset; B = bulbar; FTD = frontotemporal dementia; LL = lower limb; SOO = site of onset; UL = upper limb.

The p.I383V mutation in *TARDBP* was initially reported in a female FALS patient in 2008 (Rutherford et al., 2008). In our study, we retrospectively analyzed all reported ALS patients with p.I383V mutation and found a total of 13 cases (Table 3) (Caroppo et al., 2016; Cheng et al., 2016; González‐Sánchez et al., 2018; Lattante et al., 2012; Rutherford et al., 2008; Soong et al., 2014; Ticozzi et al., 2011; van Blitterswijk et al., 2012; Wang et al., 2020). As we know, the subtypes of FTD include behavioral variant of FTD (bvFTD), semantic dementia (SD), and progressive nonfluent aphasia (PNFA). As a variant of primary progressive aphasia (PPA) (Gorno‐Tempini et al., 2011), SD is also designated semantic variant primary progressive aphasia (svPPA) recently. Among 13 ALS patients with p.I383V variant in literatures, 10 cases had family histroy of ALS or FTD. Furthermore, in the above 10 ALS cases, 2 patients developed bvFTD before or after ALS (Caroppo et al., 2016; Cheng et al., 2016), one patient was reported alongside FTD but no concrete description (Soong et al., 2014), and one patient had no FTD but his older sister with p.I383V mutation presented svPPA (González‐Sánchez et al., 2018) (Table 3). However, in the three reported ALS patients with p.I383V mutation but without family histroy of ALS or FTD, no FTD were found before or after ALS (Table 3) (Lattante et al., 2012; van Blitterswijk et al., 2012; Wang et al., 2020). Thus, patient 3 in the present study is the first reported SALS case with p.I383V mutation presenting ALS alongside FTD, with the svPPA type.

**TABLE 3:** Clinical features of ALS patients with p.I383V mutation in TARDBP

	Gender	AOO (years)	SOO	FTD	Duration (months)	Family history	Ethnicity	Reference
1 patient	F	59	UL	No	**—**	Yes	Caucasian	(Rutherford et al., 2008)
3 patients	M	66	B	No	42	Yes	Caucasian	(Ticozzi et al., 2011)
	M	25	LL	No	**—**	Yes	Caucasian	
	F	57	UL	No	50	Yes	Caucasian	
2 patients	F	59	LL	No	>103	No	Dutch	(van Blitterswijk et al., 2012)
	M	46	LL	No	64	Yes	Dutch	
1 patient	**—**	**—**	**—**	No	**—**	No	Italian	(Lattante et al., 2012)
1 patient	**—**	**—**	**—**	Yes^a^	**—**	Yes	Chinese	(Soong et al., 2014)
1 patient	M	65	S	Yes^b^	36	Yes	French	(Caroppo et al., 2016)
2 patients	F	38	LL	No	>24	Yes	Chinese	(Cheng et al., 2016)
	F	62	B	Yes^c^	>60	Yes	Chinese	
1 patient	M	52	S	No^d^	>36	Yes	Spanish	(González‐Sánchez et al., 2018)
1 patient	F	45	UL	No	36	No	Chinese	(Wang et al., 2020)
Patient 2	F	53	B	No	23	No	Chinese	This study
Patient 3	M	57	LL	Yes	>61	No	Chinese	This study

^a^ No detailed description of FTD.

^b^ Onset of behavioral variant of FTD (bvFTD) at age 65 then ALS at age 67.

^c^ She presented bvFTD 2.5 years later.

^d^ His older sister with p.I383V mutation showed semantic variant primary progressive aphasia (svPPA).

**Abbreviations: —** = absent; AOO = age of onset; B = bulbar; FTD = frontotemporal dementia; LL = lower limb; S = spinal; SOO = site of onset; UL = upper limb.

Despite the common TDP‐43 pathology, ALS and FTD also have a genetic overlap. Although *C9orf72* is the most important overlap gene, *TARDBP* is also a critical factor for ALS and FTD. In FTD patients with *TARDBP* mutations, bvFTD is the most frequent phenotype (Caroppo et al., 2016). However, seven svPPA cases with four *TARDBP* mutations, p.G295Sp, p.A382P, p.A382T, and p.I383V, were also reported in literatures (Benajiba et al., 2009; Caroppo et al., 2016; Cheng et al., 2016; Floris et al., 2015; Gelpi et al., 2014; González‐Sánchez et al., 2018). Among them, one patient with p.G295S mutation developed bulbar‐onset ALS (Benajiba et al., 2009), one patient with p.A382P mutation developed spinal‐onset ALS (Caroppo et al., 2016), but one patient with p.A382T variant and four patients with p.I383V mutation had no ALS alongside (Caroppo et al., 2016; Cheng et al., 2016; Gelpi et al., 2014; González‐Sánchez et al., 2018) except the family members with ALS (Cheng et al., 2016; González‐Sánchez et al., 2018). Thus, patient 3 in our study is also the first svPPA case with p.I383V mutation developed ALS, which expanding the phenotype spectrum of p.I383V mutation beyond pure svPPA. Furthermore, patient 3 presented symptoms of svPPA at 57 years old, similar to the onset ages of four svPPA cases with p.I383V mutation (51 to 64 years old) in literatures (Caroppo et al., 2016; Cheng et al., 2016; Gelpi et al., 2014; González‐Sánchez et al., 2018). In addition, patient 3 in this study displayed severe blade‐like atrophy and hypometabolism in the temporal lobe by a PET/MR hybrid, which further supported the diagnosis of svPPA.

It is demonstrated that TDP‐43 contains an N‐terminal domain, two RRM RNA‐binding motifs and a C‐terminal region, which is essential for binding to heterogeneous nuclear ribonucleoproteins (hnRNPs) and splicing inhibition. Almost all *TARDBP* mutations (including p.N378D and p.I383V mutations) occur in exon 6, which encodes the highly conserved C‐terminal region of TDP‐43. Thus, further functional analysis of p.N378D and p.I383V mutations is important to investigate the pathogenesis, but unfortunately was not carried out at present.

In conclusion, our results demonstrate a low frequency of *TARDBP* mutations and expand the phenotype spectrum of *TARDBP* mutations in Chinese SALS cases. For early onset SALS patients and those comorbid FTD, genetic analysis is strongly recommended. Moreover, shared *TARDBP* mutations between ALS and FTD may aid the development of effective therapeutic strategies.

## CONFLICT OF INTEREST

On behalf of all authors, the corresponding author states that there is no conflict of interest.

## AVAILABILITY OF DATA AND MATERIALS

The dataset used and analyzed within this article will be made available from the corresponding author on reasonable request.

## AUTHOR CONTRIBUTIONS

Study design, critical revision of the manuscript, and supervision of the project were performed by Xusheng Huang. Genetic and clinical data of ALS patients were acquired by Feng Feng, Hongfen Wang, Zhanjun Wang, Xiaoyong Tao, Zhengqing He, and Fei Yang. ^18^F‐FDG PET/MR examination was carried out by Jiajin Liu and Baixuan Xu. The first draft of the manuscript was written by Feng Feng and reviewed by Hongfen Wang and Kun Zhao.

## Supporting information

Supporting Information

Figure S1
