# Supplementary material for: Genetic and clinical features of Chinese sporadic amyotrophic lateral sclerosis patients with TARDBP mutations
Source: Brain Behav. 2021 Aug 1;11(8):e2312. doi: 10.1002/brb3.2312 (PMC8413724; doi:10.1002/brb3.2312)

# Supplementary Table

**Table S1. Demographic and clinical features for total SALS patients**

|  | **Spinal onset (n=314)** | **Bulbar onset (n=77)** | ***p*-value** |
| --- | --- | --- | --- |
| **AOO (y)** | **51.9±10.5** | **56.1±11.0** | **0.002** |
| **Gender (M/F)** | **200/114** | **35/42** | **0.003** |

AOO = age of onset. A two-sample t-test was used to compare ages of onset, and a chi-square test was used to compare gender ratios.

**Table S2. Demographic and clinical features for each diagnostic category of SALS patients**

|  | **Clinically definite**  **ALS (n=228)** | **Clinically probable**  **ALS (n=109)** | **Clinically possible**  **ALS (n=54)** |
| --- | --- | --- | --- |
| **AOO (y)** | **52.1±10.7** | **56.0±10.0** | **53.1±11.8** |
| **Gender (M/F)** | **131/97** | **73/36** | **31/23** |
| **SOO (S/B)** | **178/50** | **91/18** | **45/9** |

AOO = age of onset; B = bulbar; S = spinal; SOO = site of onset

# Supplementary Figure

**Figure S1.** Detected p.N378D and p.I383V mutations in *TARDBP* gene.


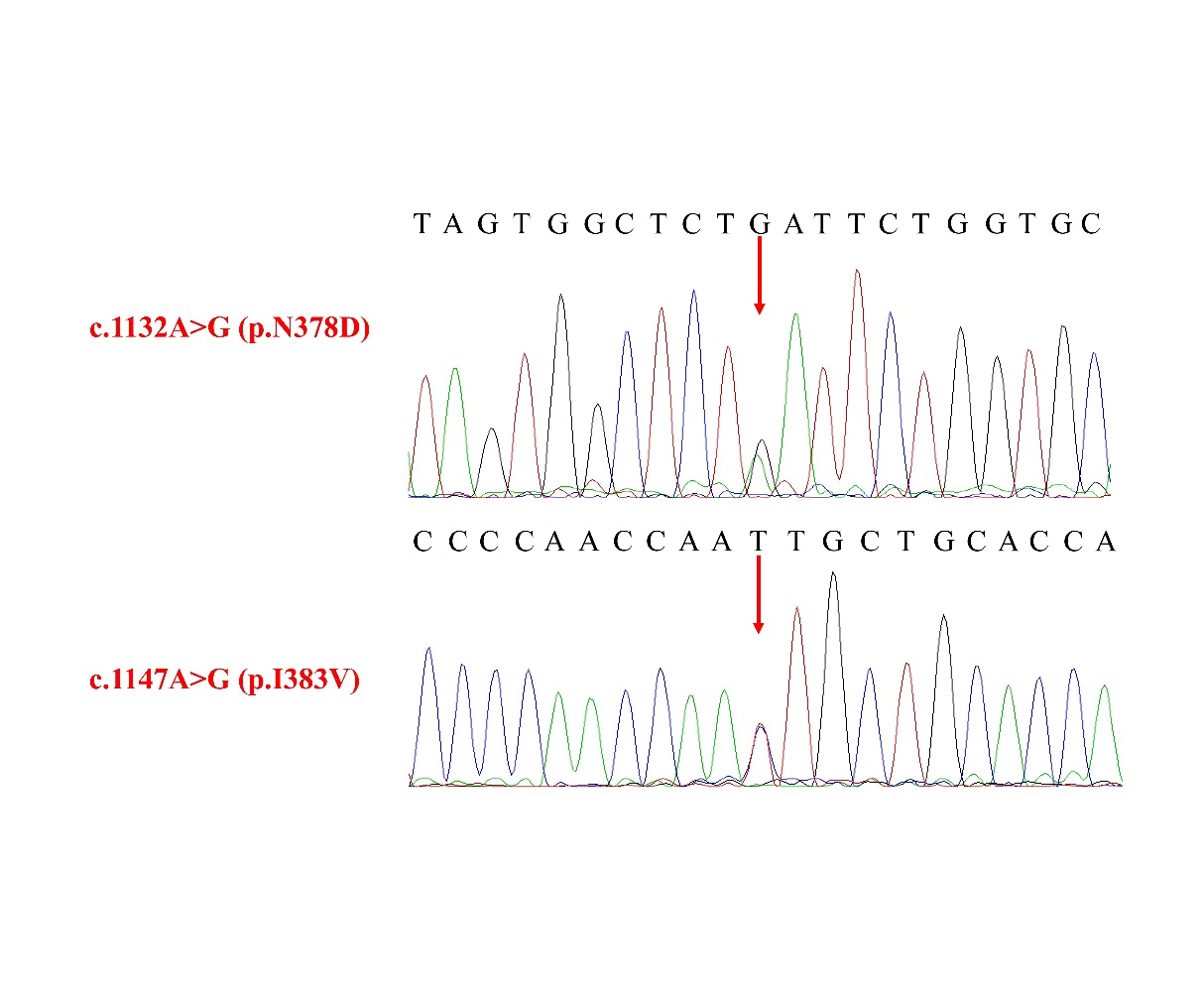

Supplement: Supplementary file 1 — Supporting Information [file BRB3-11-e2312-s001.docx]
