# Supplementary figures and images for: Genetic and clinical features of Chinese sporadic amyotrophic lateral sclerosis patients with TARDBP mutations
Source: Brain Behav. 2021 Aug 1;11(8):e2312. doi: 10.1002/brb3.2312 (PMC8413724; doi:10.1002/brb3.2312)

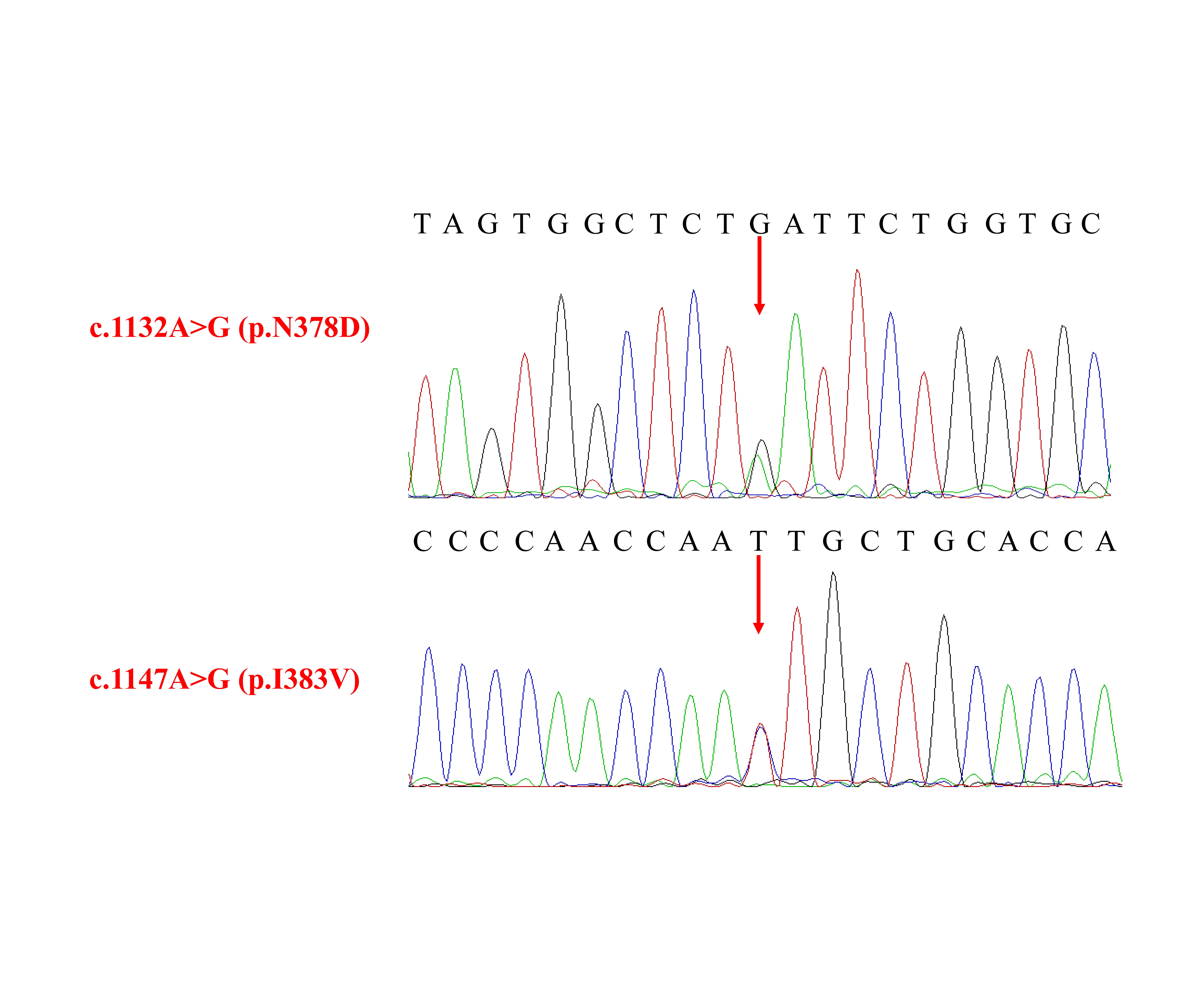

Supplement: Supplementary file 2 — Figure S1 [file BRB3-11-e2312-s002.jpg]
